# Access to Treatment for Diabetes and Hypertension in Rural Cambodia: Performance of Existing Social Health Protection Schemes

**DOI:** 10.1371/journal.pone.0146147

**Published:** 2016-01-27

**Authors:** Maryam Bigdeli, Bart Jacobs, Chean Rithy Men, Kristine Nilsen, Wim Van Damme, Bruno Dujardin

**Affiliations:** 1 Alliance for Health Policy and Systems Research, World Health Organization, Geneva, Switzerland; 2 Deutsche Gesellschaft für Internationale Zusammenarbeit (GIZ), Phnom Penh, Cambodia; 3 Chean and Jaco consulting company, Phnom Penh, Cambodia; 4 Department of Social Statistics and Demography, University of Southampton, Southampton, United Kingdom; 5 Department of Public Health, Institute of Tropical Medicine, Antwerp, Belgium; 6 Centre de Recherches en Politiques et Systèmes de Santé, Santé Internationale, School of Public Health, Université Libre de Bruxelles, Brussels, Belgium; University of Tolima, COLOMBIA

## Abstract

**Background:**

Non-communicable diseases (NCD) pose challenges to Cambodia’s health system. Medicines for NCD are on the National Essential Medicines List but no clinical guidelines support their utilization. Two social health protection schemes aimed at the informal sector population exist (Health Equity Funds and Insurance) together with two disease-specific interventions (a Peer Educator Network and Chronic Diseases Clinics) targeted at NCD patients. This study examines performance of these various schemes in relation to NCD.

**Methods:**

Cross-sectional household survey among 709 individuals self-reporting diabetes and/or hypertension in three geographical locations in rural Cambodia using a structured questionnaire investigating diagnostic and treatment pathways, health seeking behaviour, health expenditures, and financial coping mechanisms.

**Results:**

Two third of respondents with NCD were female and 55% did not belong to any scheme. The majority (59%) were diagnosed in the private sector and only 56% were on allopathic treatment that was mainly sought in the private sector (49%). Outpatient treatment cost was higher in the private sector and when using multiple providers of care. The majority were indebted, 11% due to health-related expenses. Contrary to social health protection schemes, disease-specific interventions offered better access to allopathic treatment and provided medicines in accordance with NEML.

**Conclusion:**

The benefit packages of existing social health protection schemes and services in the public health sector should be adjusted to cater for the needs of people living with NCD in rural Cambodia. Initiatives that offer active disease management strategies and promote patients and community participation appear more successful in increasing treatment adherence and decreasing the risk of financial hardship.

## Introduction

Chronic, non-communicable diseases (NCD) are a leading cause of mortality worldwide [1, 2]. The situation is not dissimilar in Cambodia, a low-income country that undergoes the epidemiological transition with emerging prominence of NCD. A cross-sectional survey conducted in 2005 found a diabetes prevalence of 5–11% amongst adults while 12–25% had hypertension, depending on the place of residence [3]. A survey in 2010, using a much larger sample, found a prevalence of 3.1% and 12.3% respectively [4]. Of patients with diabetes, 52% had no treatment while the figure for hypertension was 58%. Of diabetes patients on treatment, 58% remained uncontrolled for blood sugar level while 39% of the treated hypertensive patients had no blood pressure measurements [4].

Non-ommunicable Diseases pose special challenges to health systems geared towards managing infectious diseases and mother and child health services [5–8]. A recent literature review concluded that the health system response to NCD in the Asian Pacific Region is weak and recent at best [9]. Nonetheless, NCD feature prominently as a cause of catastrophic and impoverishing health expenses [10–12]. These costs arise from the requirement for continuous treatment over extended periods with cumulative expenses [13]. Medicines constitute a substantial proportion of these expenses [13–15]. It should be noted that these surveys do not take into account the costs resulting from foregone treatments and consequent comorbidities and complications.

To protect people with NCD from financial hardship, effective social health protection schemes are called for [13] but results to date appear mixed. A study using survey data gathered during 2002–03 from 35 countries found no financially protective effect of health insurance, nor did enrolment in such scheme improve access to treatment [15]. In India, a government insurance scheme for the poor provided negligible financial protection for NCD as it only covered costs of inpatient admissions but not of outpatient consultations. In Vietnam, health insurance was found to offer limited financial protection–due to co-payments [12]. China provides for a curious case with reports of increased out-of-pocket expenditures because of insurance [16, 17] co-existing with protective rural insurance schemes [18]; the latter were recently revised to accommodate NCD. In East Asia, subsidised public health services were found not to offer financial protection in Bangladesh [19], similar to findings from India [14]. None of these studies, however, assessed whether the patients accessed appropriate treatment.

Cambodia is known for innovative health financing schemes aimed at improving access to care and providing financial protection for the poor. They were, however, not designed with the burden of NCD in mind and therefore, it is unclear whether they offer any protection for people living with diabetes and hypertension (hereinafter termed NCD patients). This study seeks to answer this question.

## Background

Public health services are organized around the Operational Health District (OD) Model through two levels comprising districts and the province. Health districts cover 100,000–200,000 people and are made up of health centres that cover 10,000–20,000 people and deliver a minimum package of activities (MPA) consisting of preventive services and basic curative care. They are backed by the referral hospital that provides a complementary package of activities (CPA), further classified according to their ability to conduct major surgery. In practice, Cambodia’s health sector is dominated by an unregulated private market that is the preferred choice for treatment [20–22] but constitutes a major source of out-of-pocket expenditures [21, 22]. The 1996 Charter on Health Financing endorsed the introduction of nominal user fees in the public health sector [23] although medicines listed on the National Essential Medicines List (NEML) are available free of charge at the point of care. Cambodia’s approach to managing diabetes and hypertension is described in the Second National Strategic Plan for the Prevention and Control of Non-communicable Diseases [24] and in the guidelines for MPA and CPA at health centre and hospital levels respectively [25, 26]. At health centre level, services for diabetes consist of screening, health education, counselling and referral to diabetes clinics for diagnosis and treatment. These services are also recommended for patients with high blood pressure, including treatment of simple non-complicated high blood pressure with referral of complicated cases to higher levels of care. However, there are no clinical guidelines for such treatment at both levels, except for a number of specialised clinics. Medicines for treatment of diabetes and hypertension at referral hospitals are however included in the NEML [27] although no clinical guidelines support their prescription, dispensing and use.

As mentioned, Cambodia harbours numerous health financing interventions that are geared towards improving access to health services. Of notable interest, and included in this study, are health equity funds and community-based health insurance. While these health financing schemes do not specifically focus on NCD patients, disease specific interventions such as peer education networks and chronic disease clinics are also piloted in the country and specifically target NCD patients.

Health Equity Funds (HEF) are the main social health protection mechanism in Cambodia, covering around 2.6 million people or 93% of the estimated poor people in 2013 [28]. It is a third party mechanism that reimburses user fees of public health providers for services rendered to eligible poor. The latter are identified nationwide at regular intervals through proxy means testing in accordance with guidelines of the Ministry of Planning [29]. Following this community-based identification process, eligible poor receive an identification card that provides them with fee waivers at public health facilities. At the time of our study, the HEF operated at 49 hospitals and 458 health centres while they have now been extended nationwide [28].

Community Based Health Insurances (CBHI) are voluntary non-for-profit health insurance schemes that operate in selected locations in Cambodia. Beneficiaries of CBHI are entitled free health services at the point of care at contracted public health facilities. Currently CBHI covers 95,000 people, less than 1% of the Cambodian population (Personal communication with Isabel Haletz, Social Health Protection Association, December 2014, Phnom Penh Cambodia). There are no specific provisions for chronic conditions in the benefit packages of HEF and CBHI [29, 30]; patients covered by these schemes receive the same services as the general population consulting public health facilities.

At the time of our study, the peer-education network (PEN) was operated by a Cambodian NGO, MoPoTsyo [31, 32], although it has recently organized to hand-over its operations to Operational Districts in selected locations. Its peer educators are trained concerning their condition and their role and provided with basic equipment and supplies. They organise patient access to services provided by the NGO, receive lab test results and explains them to patients if required. The NGO’s role is to coach peer educators, organise lab tests and medical consultations, and facilitate access to medicines through a Revolving Drug Fund. For the latter the NGO procures generic medicines in bulk, based on a pre-defined list, and contracts private pharmacies to sell these with a 15% profit margin. Members pay for all services, although the NGO management ensures that the cost of services and products are lower than prevailing market prices. In 2012, 12496 people were registered with the network [33].

Chronic Diseases Clinics (CDC) were initiated in Cambodia by an international NGO as an integrated care initiative for people living with HIV, diabetes and hypertension [34, 35]. They operate only in selected provincial hospitals in 5 provinces. These clinics benefit from additional staff members trained in management of NCD, including medical doctors, counsellors and nurses, as well as improved infrastructure and equipment. Patients receive a unique identifier and medical file after paying a registration fee (US$1). Initially, they paid a fixed fee for consultation, medicines (3-month course) and diagnostics, with amount of fees differing according to treatment (metformin or glibenclamide) [35]. However, medicines became free of charge after 2005 [35].

A summary of coverage, entitlements and financing under each scheme is provided in Table 1.

**Table 1:** Description of schemes and respective entitlements for people with diabetes and hypertension.

	Community-Based Health Insurance	Health Equity Funds	Chronic Disease Clinic	Peer Educator Network	No scheme
**Coverage**	Voluntary enrolment with premium payment	All people living below poverty line	Passive coverage based on attendance, registration fee	Voluntary enrolment through peer educator network, enrolment free of charge	All Cambodians
**Entitlements for diabetes and hypertension**	Outpatient consultation and 3 days of medicines in public facilities, hospitalisations in public facilities	Outpatient consultation and 3 days of medicines in public facilities, hospitalisations in public facilities	Consultations, medicines, diagnostics in selected public hospitals	Support to disease management through peers, consultations, diagnostics in public facilities, medicines in private facilities contracted in RDF	Outpatient consultation and 3 days of medicines in public facilities, hospitalisations in public facilities
**Financing**	Insurance premium. Services free of charge at point of care	Services free of charge	Fee for package of consultation, diagnostic and 3-months course of medicines	Fee for service, including for medicines	Fee for service at point of care, medicines free of charge

## Methods

Ethical approval was obtained from the Cambodian National Ethics Committee for Health Research (0008 NECHR) and the Research Ethics Review Committee of the World Health Organization (RPC 551). This study is part of a broader research project entitled *“Access to medicine for chronic non-communicable diseases in rural Cambodia*: *synergising the district health system*, *risk protection schemes and socio-cultural factors”*. Written informed consent was obtained from all study participants during the screening and the interviews. The conceptual framework adopted in this research project is put forward by Bigdeli et al [36] and applies a health system perspective to examine determinants of access to medicines at both demand and supply side. For such in-depth investigation, we adopted a mixed methods approach [37, 38], combining quantitative and qualitative data collection from grey and published documents, a household survey, a facility survey, and in-depth interviews with patients and key informants at district and central level.

This paper reports on the results of the household sample survey, which provides information on access to medicine for NCD patients who are members of social health protection schemes or specific interventions for NCD, financial protection and coping mechanisms. Individuals eligible for inclusion in the sample had to be at least 25 years old, have a clinical diagnosis of diabetes and/ or hypertension.

We sampled from Baray Santuk OD in Kampong Thom province, Kirivong OD in Takeo province and Samrong OD in Oddar Meanchey province. These three ODs were purposively selected because of the presence of social health protection schemes for the rural informal sector population (HEF and CBHI) and/or disease-specific intervention (CDC in the nearest provincial hospital and PEN).

To estimate the sample size according to each OD, the following formula was applied:

$$n=\frac{t^{2}p(1-p)}{m^{2}}\times D$$

Whereby n is required sample size, t is 1.96, the critical value for confidence level at 95%, p the estimated diabetes prevalence at 4.5% (0.045), m the margin of error at 3.75% (standard value of 0.0375) and D the design effect (standard value of 2.0).

The formula yielded a sample size of at least 235 individuals per OD or 705 individuals in total.

Because there is no list or registry of individuals with diabetes and hypertension, they cannot be selected directly. To identify individuals eligible for the survey, a rapid screening tool was developed querying adults in each household concerning awareness of disease status for diabetes and/or hypertension and enrolment with any of the concerned schemes (CBHI, HEF, PEN, CDC or None). Using the below formula, it was estimated that 15,653 individuals had to undergo the rapid screening to yield a sample size of 704 individuals:

$$N=\frac{n}{p}$$

whereby N is the total number of individuals required for rapid screening, n is sample size of individuals with diabetes/hypertension and p the estimated diabetes prevalence at 4.5% (0.045).

The prevalence rate used for sample size calculation was the prevalence of hypertension among CBHI members in Kirivong OD, estimated at 4.5% based on CBHI members records; this prevalence is considered conservative according to King et al [3]. The number of individuals to be approached for the rapid screening was therefore rounded down to 15,000 in total or 5,000 per OD. Assuming that each household contained two adults, the number of households to be targeted was 7,500 in total or 2,500 per OD.

The sample of households was selected using two stages cluster sampling. In the first stage, 62 villages in Baray Santuk OD and 60 villages in both Kirivong and Samrong ODs were selected with probability proportional to the size of population in the village. In the second stage, 41 households per village were selected using Simple Random Sampling, for approximately 2,500 households per OD. Accounting for attrition, the final sample for the rapid screening was composed of 7,361 households. Based on the results of the rapid screening questionnaires, 1,444 individuals were found to fulfil the criteria for sample inclusion of which 194 individuals identified with diabetes or diabetes/hypertension and 1,250 with hypertension.

Among these individuals, 750 NCD patients were selected based on disease status. All individuals with diabetes only or diabetes and hypertension were selected to take part of the survey. In the case of hypertension only, a subsample of individuals was selected using a two phases sampling design as follows. First, the 1250 identified patients with hypertension were stratified by OD and by social health protection scheme. Then, 50% of these individuals were selected in each stratum using Simple Random Sampling. The final size of the sample of hypertensive patients were 556 individuals.

Of the 750 individuals with diabetes diabetes/hypertension and hypertension, 709 individuals completed the survey. The remainder 41 were not at their place of residence when visited and no efforts were made to contact them again.

To account for sampling design, three weights were calculated.

Weight *w*_1*i*_ corresponds to the first stage of selection:

$$w_{1i}=\frac{X}{X_{i}\times n_{i}}$$

whereby X is the total number of households per village in a given OD, X_i_ is the number of households in village and n is the number of villages selected in a given OD.

Weight w_2i_ corresponds to the second stage of selection:

$$w_{2i}=\frac{X_{i}}{41}$$

whereby X_i_ is the number of households in the villages selected in village and 41 is the number of households selected per village.

The weight w_3_ accounts for the selection of hypertensive patients:

$$w_{3}=\frac{1}{0.5}$$

whereby the weight is equal to one over the sampling fraction, which is 50% irrespective of strata.

Because all individuals with diabetes only and patients with diabetes and hypertension were included in the final phase of sampling, only the first two weights were applied to these groups. The final weight is the product of the three weights calculated and is the same for individuals in a given OD and strata. The screening happened in May 2013 while structured interviews took place in July-August of the same year.

The 709 individuals were interviewed by qualified researchers, following training in concepts of NCD and use of a questionnaire using a mix of pre-coded structured and open-ended questions. The questionnaires were pre-tested and queried about issues related to diagnoses, treatment, reasons for consulting the type of frequented health provider, health seeking behaviour and associated expenditure for outpatient consultations in the 30 days prior to interview and hospitalization in the last 12 months, sources of money and coping mechanisms for dealing with the costs of their illness. Respondents were also questioned on household’s debt, including self-reported formal and informal credits incurred at household level as defined previously by Ir et al in the Cambodian context [39]. Respondents were asked whether they took traditional medicines obtained through the Khmer traditional healers; other treatments received in clinics, pharmacies, health centres and hospitals were categorized as “allopathic”, Furthermore, data collectors recorded the names of medicines used by respondents for diabetes and hypertension. Recorded medicines names were later converted in their International Non–proprietary Name (INN) and compared to the NEML; the medicines were coded as “according to NEML” if they were on that list. If the medicine was not on the list, it was coded as “not on NEML” and if the interviewee was unable to remember or show the medicine, this was coded as “don’t know”.

Open-ended questions were pre-coded before data entry. Data were entered in an MS Excel database and transferred to Stata version 13.1 for analysis. Respondents were primarily stratified according to (non)enrolment in schemes. Statistical comparisons were made when relevant, given the fundamental differences between schemes intended to provide financial protection (HEF and CBHI) and those intended to provide diagnostic and treatment (PEN and CDC). *p-values* are reported for Fisher’s exact test, significance determined at 5% level.

## Results

### Sample characteristics

A total of 7,361 households were approached during the screening process; 1559 individuals reported suffering from hypertension or diabetes; 1,357 (18%) adults had hypertension, 120 (2%) diabetes and 82 (1%) both conditions. Of the 1,559 interviewees reporting with one or both of the concerned conditions, 53% were enrolled with one or more scheme: 18% (281) HEF, 13% (198) CDC, 9% (134) CBHI, 7% (109) PEN and 7% (108) multiple schemes.

The subsequent total number of interviewees was 709: 78% (558) with hypertension, 14% (97) with diabetes and 8% (54) with both conditions. Fifty percent did not belong to any scheme (Table 2). Their mean age was 56 years and two thirds were female. The majority were diagnosed more than 3 years prior to interview.

**Table 2:** Sample characteristics.

	CBHI	HEF	CDC	PEN	NS	Total
*N*	*73*	*134*	*113*	*34*	*355*	*709*
*%*	*10%*	*19%*	*16%*	*5%*	*50%*	*100*
**Province**						
Baray Santuk	0	31%	87%	60%	32%	39%
Samrong	5%	24%	6%	33%	24%	19%
Kirivong	95%	45%	7%	7%	44%	42%
**Mean age** (SD)	54 (12)	59 (16)	56 (12)	56 (10)	55 (13)	56 (13)
**Gender**						
Male	28%	35%	43%	25%	34%	34%
Female	72%	65%	58%	75%	66%	66%
**Time since diagnosis**						
< 1 year	18%	18%	8%	7%	7%	15%
1 year	24%	17%	11%	18%	28%	21%
2 years	12%	15%	21%	30%	16%	16%
3 years	14%	18%	15%	14%	12%	14%
4+ years	32%	32%	45%	30%	36%	34%

**CBHI** = community-based health insurance; **CDC** = Chronic disease clinic; **HEF** = health equity fund; **NS** = no scheme; **PEN** = peer education network; **SD** = standard deviation

### Diagnostic and treatment pathways

As can be seen from Table 3, the schemes presented significant differences as to the place of diagnostic (p<0.001): the majority of respondents who did not belong to any scheme and those attending CDC were diagnosed in the private sector (68% and 61% respectively). In comparison, fewer members of CBHI, HEF or PEN schemes were diagnosed in the private sector (41%, 36% and 31% respectively).

**Table 3:** Diagnostic and treatment pathways^§^.

	CBHI	HEF	CDC	PEN	NS	Total	*p value*
*N*	*74*	*134*	*113*	*34*	*355*	*709*	
**Place of diagnosis**							<0.001
PEN	1%	1%	1%	39%	1%	3%	
Public	41%	36%	31%	13%	27%	30%	
Private	50%	56%	61%	44%	68%	61%	
Other	8%	7%	7%	4%	5%	6%	
**Current treatment**							<0.001
Allopathic	32%	48%	57%	85%	51%	51%	
Traditional	9%	12%	5%	5%	8%	8%	
No treatment^†^	59%	40%	37%	10%	41%	41%	
**Usual place of treatment** ^§^ *(N)*	*26*	*70*	*71*	*32*	*200*	*399*	*<0*.*001*
Public facilities	35%	23%	14%	7%	21%	20%	
Private clinic	32%	33%	62%	7%	46%	43%	
Private pharmacy^¶^	0%	13%	7%	4%	11%	9%	
PEN	2%	1%	0%	47%	0%	4%	
Multiple sources	31%	30%	15%	35%	18%	22%	
Other	0%	0%	1%	0%	3%	2%	

^†^No treatment includes people who declared receiving no treatment and those who report dropping out of their treatment

^§^ Figures reported here are for people on allopathic treatment only

^¶^ Includes formal or informal drug shops

The proportion of people receiving allopathic treatment at the time of interview varied significantly between schemes (p<0.001). Interviewees covered by PEN and CDC were more likely on allopathic treatment (85% and 57% respectively), than HEF beneficiaries (48%) or CBHI members (32%). The proportion of interviewees without treatment at the time of interview was 40%, with the highest figure amongst CBHI members (59%) and the lowest amongst PEN members (10%). Reasons for dropping out of treatment were multiple and included beliefs that the disease was not serious, feeling better and financial constraints (figures not shown in table). In total, only 8% across schemes reported taking traditional medicines.

A little less than a quarter of interviewees used multiple sources of treatment (22%) Private providers, both formal and informal, were a frequent source of treatment for all respondents (43%). However, people covered under CDC (62%) and people not belonging to a scheme (46%) used private providers more frequently than HEF, CBHI and PEN beneficiaries (32%, 33% and 7% respective, p<0.001). Of those covered by HEF and CBHI, a third or less got treatment from public facilities while only 14% of CDC patients reportedly did, despite the fact that CDC clinics belong to the public sector.

In all five groups, the two main reasons for not having treatment were feeling better (49%) or being unable to afford treatment (45%). For those who never received any treatment, insufficient knowledge about their disease and its outcome was the main reason and 44% of them reported they would seek treatment in the future. (figures not shown in table)

There was a significant difference of care seeking attitude between people who chose public and private health services (p<0.001).As presented in Table 4, Fifty-two percent of respondents chose PEN services because these were covered by the scheme they belong to. On the other hand, trust in provider was the main driver of choice for people seeking care in private clinics (53%) and private pharmacies (42%). Quality of services and medicines were reported as core elements of trust (figures not shown in table).

**Table 4:** Reason for choosing provider.

	Public services	Private clinics	Pharmacy	PEN	Multiple providers	Other	Total	*p value*
*N*	*80*	*171*	*36*	*16*	*88*	*8*	*399*	*<0*.*001*
Covered by scheme	9%	1%	0%	52%	1%	23%	5%	
Been there before	16%	16%	15%	5%	0%	0%	12%	
Trust in provider	33%	53%	42%	18%	12%	60%	37%	
Multiple reasons	37%	17%	16%	26%	83%	17%	36%	
Other	4%	13%	26%	0%	4%	0%	10%	

A third of cases reported to take more than one medicine to treat their diabetes (32%) or their hypertension (28%). As shown in Table 5, for diabetes 62% of medicines were in accordance with NEML. The figure for hypertension was 45%. In both groups, a large number of respondents could not produce the labels of the concerned medicines or did not remember the name of the medicine they were taking. There was no significant difference between schemes regarding accordance of diabetes or hypertension medicines to NEML.

**Table 5:** Medicines used^§^^.^.

	CBHI	HEF	CDC	PEN	NS	Total	*P value*
Main diabetes medicine *(N)*	*11*	*9*	*18*	*34*	*49*	*120*	*NS*
On NEML	40%	70%	44%	73%	63%	*62%*	
Not on NEML	20%	16%	0%	6%	0%	8%	
Don’t know ^‡^	40%	14%	56%	21%	27%	30%	
Main hypertension medicine *(N)*	*17*	*60*	*50*	*30*	*169*	*326*	*NS*
On NEML	58%	37%	34%	54%	49%	45%	
Not on NEML	17%	31%	34%	26%	32%	31%	
Don’t know^‡^	24%	32%	32%	20%	19%	24%	

^**§**^Figures reported here are for people on allopathic treatment only

^‡^ People could not produce the medicines they took or did not know the name

**NEML** = National Essential Medicines List

### Treatment costs

People without scheme coverage and those covered by HEF incurred the highest median medical costs for their last OPD visit (US$ 6) followed by people enrolled with CDC (US$ 5) (Table 6). Costs for beneficiaries of CBHI and PEN were lower: US$ 4 and 3 respectively. Except for CBHI members, medicines represented a large proportion, ranging from 60 to 100%.

**Table 6:** Cost of last outpatient consultation in US Dollars ^§^.

	Median costs (min-max)					
	CBHI	HEF	CDC	PEN	NS	Total
*N*	*22*	*64*	*55*	*49*	*191*	*381*
**Medical costs**	4 (0–25)	6 (0–45)	5 (0–314)	3 (1–25)	6 (0–399)	5 (0–399)
Health service costs	0 (0–18)	0 (0–27)	0 (0–115)	1 (0–6)	0 (0–110)	0 (0–115)
Medicines costs	0 (0–25)	4 (0–30)	5 (0–200)	2 (0–22)	4 (0–349)	4 (0–350)
Diagnostic costs	0 (0–1)	0 (0–15)	0 (0–13)	0 (0–7)	0 (0–50)	0 (0–50)
**Transport costs**	1 (0–5)	0 (0–45)	0 (0–100)	1 (0–13)	1 (0–200)	0 (0–200)
Cost of medicines as % of direct OPD costs	0	63%	100%	70%	60%	71%

^§^Figures reported here are for people on allopathic treatment only

Only 18 respondents in the sample reported a hospitalisation for the concerned conditions in the 12 months prior to interview. The median total cost of hospitalization was US$ 41 (0–249). Medicines represented 21% of this cost. The small number of observations did not allow for disaggregating these results by scheme. (Results not shown in table)

Table 7 provides a comparison of cost incurred during the last OPD visit according to place of care. The median medical cost was higher at private clinics (US$8) and private pharmacies (US$4) than public facilities (US$1) and PEN (US$3). Interviewees reporting to have frequented multiple providers also incurred higher cost (US$6). The few respondents grouped under ‘others’ include those who sought care abroad (Vietnam, Thailand) which explains their high transport costs.

**Table 7:** Treatment costs in US Dollars by source of treatment for the last outpatient visit ^§^.

	Median costs (min-max)						
	PEN	Public Facility	Private clinic	Pharmacy	Multiple sources	Other	Total
*N*	*25*	*73*	*149*	*38*	*89*	* 7*	*381*
**Medical costs**	3 (1–16)	1 (0–399)	8 (0–314)	4 (1–26)	6 (0–175)	48 (8–68)	5 (0–399)
Health services	1 (0–6)	0 (0–18)	0 (0–115)	0 (0–20)	0 (0–37)	0 (0–5)	0 (0–115)
Medicines	2 (0–15)	1 (0–349)	5 (0–200)	3 (0–25)	5 (0–155)	18 (0–37)	4 (0–349)
Diagnostic	0 (0–1)	0 (0–50)	0 (0–40)	0 (0–7)	0 (0–25)	25 (0–40)	0 (0–50)
**Transport costs**	1 (0–5)	1 (0–200)	0 (0–125)	0 (0–5)	0 (0–50)	7 (1–62)	0 (0–200)
Cost of medicines as % of direct OPD costs	88%	42%	67%	71%	78%	36%	71%

^§^ Figures reported here are for people on allopathic treatment only

No statistical tests are performed to compare treatment costs as the study mixes financial protection schemes on one hand and disease-specific interventions on the other hand: while HEF and CBHI cover the cost of diagnosis and treatment and aim at protecting beneficiaries from out-of-pocket and catastrophic expenses, CDC and PEN are mainly geared at enabling affordable access to clinical services and medicines against established user fees. Therefore, statistically comparing treatment costs of the schemes and interventions is not feasible. The highest costs incurred by HEF beneficiaries is however counter-intuitive and will be discussed in the Discussion section.

### Coping strategies

Almost all respondents paid for their treatment from their regular source of income, often supplemented by borrowing money or by gifts by friends and relatives (Table 8). A third of HEF beneficiaries and half of CBHI members reported that their scheme covered the costs of treatment.

**Table 8:** Source of money for treatment^§^^∞^.

	CBHI	HEF	CDC	PEN	NS	Total
*N*	*26*	*70*	*71*	*32*	*200*	*399*
Covered by CBHI	52%	0	0	4%	0	4%
Covered by HEF	0	30%	0	4%	0	6%
Usual income	97%	76%	92%	90%	89%	88%
Loan	17%	32%	18%	11%	15%	18%
Sold assets	3%	10%	17%	11%	8%	10%
Gift/charity	45%	24%	10%	16%	23%	22%

^§^ Figures reported here are for people on allopathic treatment only

∞ Multiple answers allowed, percentages add to >100%

Almost one in two respondents reported being in debt, with little variation according to scheme (Table 9). The highest median amount of general debt observed was for PEN (US$1,097) while the median amount of debt was similar for all other categories (US$ 499).

**Table 9:** Debt, debt for health and sources to repay.

	CBHI	HEF	CDC	PEN	NS	Total
*N*	*73*	*134*	*113*	*34*	*355*	*709*
**General debt**						
Yes	48%	53%	46%	42%	48%	48%
No	52%	47%	54%	58%	52%	52%
						
**Amount of debt (in US$)** ^∫^ *(N)*	*30*	*68*	*46*	*26*	*167*	*337*
Median	499	499	500	1097	499	499
Min	18	7	7	6	7	6
Max	10000	4000	5000	5800	35500	35500
						
**Main reason for debt**^∫^ (N)	35	70	51	14	167	337
Health reason	28%	37%	27%	11%	21%	25%
Other	72%	63%	73%	89%	79%	75%
**Source of money for payment**^∫^∞*(N)*	*35*	*70*	*51*	*14*	*167*	*337*
Sell assets/livestock/ land	51%	28%	20%	49%	42%	37%
Increase labour	20%	53%	50%	36%	30%	37%
Usual income	69%	43%	44%	48%	53%	51%
Other	11%	17%	3%	5%	10%	10%

^∫^ Figures reported only for people who declare having debt

∞ Multiple answers allowed, percentages add to >100%

Among people who reported being indebted, 25% invoked health reasons, equal to 11% of the total sample. About one third of interviewees covered by social health protection schemes were indebted for health-related expenses (37% for HEF and 28% for CBHI). These figures are similar to those enrolled with CDC (27%), but higher than PEN (11%). Sources of money to repay loans were multiple and ranged from regular income to increased labour or selling assets, livestock and land.

### Prospects

Depending on scheme affiliation, 57–69% of respondents perceived their health as worsening or had very negative expectation. Only 20% of respondents considered their financial situation as good or stable. The remainder anticipated future financial constraints, especially those covered by health financing schemes (CBHI 26% and HEF 36%) compared to people enrolled with disease specific interventions (CDC 12% and PEN 20%) or no scheme at all (17%).

## Discussion

### Sample characteristics

During the initial screening of households, 18% of adults reported having hypertension and 2% diabetes. More than half of cases with one of the concerned conditions were enrolled with a social health protection scheme or a disease-specific intervention (53% in total), much higher than observed amongst the general Cambodian population. For example, 10% of our study sample enrolled with CBHI while the respective figure for Kirivong OD (one of the study locations) is 4% (Personal correspondence, Sam Sam Oeun, Buddhism for Health, Kirivong, Takeo Province, Cambodia). As CBHI enrolment is voluntary, this seems to indicate a tendency of NCD patients to seek some form of social health protection to cover their medical needs. Consistent with other studies [40], women at the screened households were more likely to have one of the two conditions than men. While this imbalance does not have clinical explanations, Vialle-Valentin et al [40] suggest that this may be due to women accessing more appropriate care because of their reproductive health needs and caregiver role in the family, and therefore being diagnosed for chronic conditions.

### Diagnostic and treatment pathways

Despite the fact that all four schemes are geared to enable access to public health services, the private sector constituted an important source of care: the majority, 61%, were diagnosed in the private sector and 52% sought treatment at private clinics pharmacies. There was a tendency to shop around for treatment amongst multiple providers (22%), also for PEN members. These findings are consistent with those of other studies in Cambodia and elsewhere [20, 41–45]. Meessen et al [20] elaborated on the pluralistic nature of the health system in Cambodia and analysed in detail the care seeking pathways. They concluded that the private sector sometimes complements public sector services, including management of NCD, due to unavailability of medicines in the public sector.

Trust was an important element of provider choice and seemed to override benefits provided by all schemes except PEN: trust is the main reason for provider choice in 37% of households. In comparison, only 5% of respondents claim they choose their providers based on whether their services are included in the benefit package of the schemes they belong to. In other terms, when they do not trust the providers selected by the schemes they belong to, people do not hesitate to use services not covered by the scheme, such as private clinics or drug shops. As a consequence, none of the schemes, except PEN, offered a treatment package, including the disease management approaches, which would promote appropriate health seeking behaviour among their members. Porter et al [46] also mentioned lack of trust in providers as a potential barrier to management of NCD in Kenya. In order to promote consultation of public health providers it is necessary to better understand the meaning of quality of services and “quality of medicines” amongst NCD patients to develop more responsive services. Trust in providers may contribute to the observed use of multiple health care providers: the separation between public and private health service provision is an artificial divide in Cambodia. While public and private infrastructure and services are effectively separate; there is a considerable overlap of human resources with no restrictions on dual practices [44]. Osawa et al. [47] noted that Cambodian patients trust individual health workers rather than the institutions at which they are employed, and that might constitute the real determinant of health seeking. Conduct and relationships of private health providers with patients were also observed as being more aligned with cultural expectations, contrary to service at public health facilities [48, 49]. However, using private providers or shopping around for treatment implied higher medical costs (US$ 4–8 vs. US$1–3 at public sector and PEN).

The proportion of respondents reported being on allopathic treatment was51%, only slightly higher than found previously—42% for hypertension and 48% for diabetes [4]. The practice of providing only medicines for 3 days in public facilities likely contributes to the low proportion of NCD patients on allopathic treatment. Similar low access rates to medicines for NCDs in LMICs have been reported elsewhere: in a five country study, Vialle-Valentin et al. [40] found rates of 16% in Uganda to 49% in Jordan). Sarayani et al. [50] also found a low utilization of diabetes medicines in Iran.

Diabetic patients taking allopathic treatment were more likely to take medicines listed on the NEML than hypertensive patients (62% vs. 45% respectively).The high proportion of interviewees who could not name their treatment or could not produce medicines (24–30%) suggest poor information practices towards patients and lack of patient involvement in the management of their chronic condition. This situation is consistent with studies across Asia, that show different beliefs and expectations between doctors and patients as to what constitutes successful hypertension management and who is most responsible for it [51].

More patients enrolled with disease-specific schemes used allopathic treatment (60–89%) than those belonging to a health financing schemes (39–52%). The observed health financing schemes are not geared towards NCD while PEN and CDC target only patients with diabetes and hypertension. The PEN appeared to perform best: it had the highest rates of people on modern treatment (89%), with medicines in line with the NEML, especially for diabetes (74%). These results are not surprising, as Cambodian public health services offer only limited NCD services to NCD patients and health financing schemes do not have additional arrangements to enable their beneficiaries access to such services. The CDC, on the other hand, adopts a passive supply-oriented approach whereby members benefit from their entitlements only when actively using its services. The registration fee for the CDC and transport costs to such facilities at the provincial capital may be deterrents to treatment adherence. In comparison, the PEN is strongly present within the community and adopts a demand-centred approach, bringing services closer to the user. The peer educator is tasked with active screening within the community as well as disease management strategies.

### Treatment costs

Respondents incurred the same levels of out-of-pocket spending for their routine care (outpatient visits, regular diagnostic services and medicines), irrespective of scheme affiliation (median ≈ US$5).

In comparison, daily wage levels for unskilled workers are reported slightly around US$5.2 in 2015 [52] and the legal minimum wage of garment factory workers was set at US$100 per month in 2014 [53]: the cost of an episode of outpatient care therefore represents about one day of wages of an unskilled construction or garment factory worker. Interviewees without scheme affiliation had higher outlier costs of treatment (maximum to US$499), up to ten times higher than people with a scheme affiliation, suggesting vulnerability to catastrophic health expenditures, which warrants further exploration. Medicines consistently represented a high proportion of total expenditures (60–100%). Bhojani et al [14] reported similar levels in India (66.3%), findings supported by larger studies also point to the unaffordability of medicines for NCDs in LMICs [54, 55].

Access to essential drugs is one of the five priority actions by the Lancet NCD Action Group and the NCD Alliance for addressing the NCD crisis [55–57]. In Cambodia, medicines on the NEML are free of charge for patients at public health facilities. It is therefore surprising that medicines represent such high proportion of medical costs at such facilities, unless they are not available. The common 3-day treatment course at public health facilities is largely insufficient whereby patients probably refill their prescriptions at the private sector.

Additional barriers to NCD medicines at public facilities relate to inclusion of NCDs as part of service packages (i.e. MPA and CPA) together with availability and application of appropriate guidelines at different levels of the health system. When these conditions are not met, free provision of medicine at the point of care will not guarantee adequate access. As mentioned, the consequence is a preference for the private sector at the expense of public services.

Although Vialle-Valentin et al.[40] indicated that that people in LMIC with NCD have better access to NCD medicines when covered by health insurance scheme, our finding and those of other show that such health financing arrangements are not sufficient and point to the requirement for active disease management strategies to cater for the needs of people with NCD [12, 17, 18]. Rahman et al. [19] discussed that standalone financial risk protection schemes or disease management arrangements are insufficient and that they need to be combined for more effective NCD treatment outcome. Our results reinforce calls for more holistic approaches to access to care for diabetes and hypertension patients, approaches that go beyond including appropriate medicines on the NEML and centralized public procurement, but enable reconfiguration of health systems that are responsive to new challenges of NCD [57].

### Coping strategies

The proportion of interviewees indebted because of health expenses was high across schemes, 11%, much higher than found among the general population, 3.8% [58], suggesting financial vulnerability of people living with diabetes and hypertension. Chronic illness, loss of income, indebtedness and poverty are interconnected elements that constantly influence each other [59, 60].

### Limitations

We have limited our investigation to people with diabetes and hypertension without comparing with people who do not suffer from a chronic condition. The study was not designed for a formal statistical comparison between schemes and can only point towards difference between social health protection schemes and disease-specific interventions, some of which are inherent to scheme design: We did not investigate reasons for not being on treatment or dropping out, although information collected suggested costs of treatment and transport as main reasons. As this is a household survey, we did not investigate the quality of health services and appropriateness of treatments from the supply perspective. Given the recall bias, we failed to ascertain the timing of health related debts whereby they cannot necessarily be ascribed to shortcomings of the schemes as they can have been acquired beforehand. Furthermore, in the Cambodian context, people use informal credit for multiple and interconnected reasons, including health expenditures and it is difficult to separate the reasons of indebtedness and attribute debt to certain health conditions. [39]

## Conclusions

The main social health protection schemes in Cambodia offer very limited financial access to medicine for members with diabetes and hypertension in the country’s rural areas. Disease specific interventions established for this purpose perform better though to a limited extent, the peer education network having the best results. Consequently, NCD patients rely on private health service provision or shop around for treatment, with negative consequences on access and adherence to treatment, and high out-of-pocket expenditures. The nature and content of health services at public health facilities and consequently the benefit packages of social health protection schemes should be adjusted to cater for the needs of people living with diabetes and hypertension. Initiatives that offer active disease management strategies and promote patient and community participation in addition to access to general health care or medicines for NCD appear more successful in increasing adherence to treatment and decreasing financial hardship related to care seeking.
